# Initial impact and cost of a nationwide population screening campaign for diabetes in Brazil: A follow up study

**DOI:** 10.1186/1472-6963-8-189

**Published:** 2008-09-22

**Authors:** Cristiana M Toscano, Bruce B Duncan, Sotero S Mengue, Carísi Anne Polanczyk, Luciana B Nucci, Adriana Costa e Forti, Cláudio D Fonseca, Maria Inês Schmidt, the CNDDM Working Group

**Affiliations:** 1 Graduate Studies Program in Epidemiology, School of Medicine, Federal University of Rio Grande do Sul, Porto Alegre, RS, Brazil; 2 Center for Diabetes and Hypertension Studies, Federal University of Ceará, Fortaleza, CE, Brazil; 3 Brazilian Ministry of Health, Health Policy Division, Brasília, DF, Brazil

## Abstract

**Background:**

In 2001 Brazilian citizens aged 40 or older were invited to participate in a nationwide population screening program for diabetes. Capillary glucose screening tests and procedures for diagnostic confirmation were offered through the national healthcare system, diagnostic priority being given according to the severity of screening results. The objective of this study is to evaluate the initial impact of the program.

**Methods:**

Positive testing was defined by a fasting capillary glucose ≥ 100 mg/dL or casual glucose ≥ 140 mg/dL. All test results were tabulated locally and aggregate data by gender and clinical categories were sent to the Ministry of Health. To analyze individual characteristics of screening tests performed, a stratified random sample of 90,106 tests was drawn. To describe the actions taken for positive screenees, a random sub-sample of 4,906 positive screenees was actively followed up through home interviews.

Main outcome measures considered were the number of diabetes cases diagnosed and cost per case detected and incorporated into healthcare.

**Results:**

Of 22,069,905 screening tests performed, we estimate that 3,417,106 (95% CI 3.1 – 3.7 million) were positive and that 346,168 (290,454 – 401,852) new cases were diagnosed (10.1% of positives), 319,157 (92.2%) of these being incorporated into healthcare. The number of screening tests needed to detect one case of diabetes was 64. As many cases of untreated but previously known diabetes were also linked to healthcare providers during the Campaign, the estimated number needed screen to incorporate one case into the healthcare system was 58. Total screening and diagnostic costs were US$ 26.19 million, the cost per diabetes case diagnosed being US$ 76. Results were especially sensitive to proportion of individuals returning for diagnostic confirmation.

**Conclusion:**

This nationwide population-based screening program, conducted through primary healthcare services, demonstrates the feasibility, within the context of an organized national healthcare system, of screening campaigns for chronic diseases. Although overall costs were significant, cost per new case diagnosed was lower than previously reported. However, cost-effectiveness analysis based on more clinically significant outcomes needs to be conducted before this screening approach can be recommended in other settings.

## Background

Population, nutrition and epidemiological changes in the last century have produced a health risk profile in which chronic diseases such as diabetes mellitus account for a growing proportion of the total disease burden [1]. In 2000 an estimated 171 million people, or 2.8% of the world's population, were living with diabetes, Brazil being one of the 10 countries with the highest number [2].

Diabetes is associated with high morbidity and mortality, and substantial loss in quality of life. Associated direct medical costs vary from 2.5% to 15% of total national health expenditures, depending on prevalence and treatment availability [3]. Annual deaths caused by diabetes in Latin America and Caribbean have been estimated at 340,000 in 2000, representing a loss of 760,000 years of productive life and total costs of U$ 65 billion [4]. In the last decade, proportional mortality attributable to non-communicable diseases rose significantly in Brazil, ranking first in most states. Diabetes figures among the 10 major causes of mortality in the country [5] and current best data suggests that the prevalence of undiagnosed diabetes is high [6].

The efficacy of various treatments in reducing diabetes complications is well established [7-10]. Considering the existence of a detectable pre-clinical period and availability of acceptable and accurate screening tests, screening for diabetes seems logical. Nonetheless, benefits of early detection and treatment of undiagnosed diabetes and its economic implications have yet to be clearly demonstrated [11,12]. Thus, opportunistic screening of high-risk individuals, as opposed to population-wide approaches, has been recommended [13-15].

In this context, in 2001, the Brazilian Ministry of Health proposed a National Plan for the Reorganization of Diabetes Mellitus and Hypertension Care [16]. As part of the plan, a National Screening Campaign to Detect Diabetes Mellitus was conducted. To our knowledge, this is the first nationwide, population-based, campaign-style diabetes screening program conducted. In Brazil, no specific diabetes screening strategies were in place prior to this screening campaign.

The objectives of this report are to briefly describe the program, to evaluate its impact in terms of case detection and incorporating detected cases into medical management, to describe the total cost, and to estimate the cost per new diabetes case diagnosed.

## Methods

The main characteristics of the nationwide screening program for diabetes have been previously described [17]. Briefly, all Brazilian citizens aged 40 or older were invited via mass media mobilization strategies, to participate in the National Campaign to Detect Diabetes Mellitus, which was conducted by trained healthcare professionals working in the publicly funded national healthcare system between March 6^th ^and April 7^th^, 2001. Screening was performed using fingerstick capillary blood and portable meters. At that time, there were approximately 40,000 primary healthcare units distributed in the country's 5,507 municipalities, most of the latter managing, to a varying degree, their healthcare services locally. The number of screening tests to be performed was determined assuming that 75% of Brazilians aged 40 years or older estimated to depend exclusively on public healthcare services would participate [18].

At screening, individuals presented to primary healthcare units, answered a brief questionnaire and had capillary glucose measurement done. Age, ongoing treatment for diabetes, and fasting condition were recorded on individual forms. Those who informed having had no food ingestion in the previous 4 hours were considered to be fasting. Individuals with prior diabetes diagnosis or those who informed to be receiving diabetes therapy were not excluded from participation in the Campaign.

Referral for confirmatory diagnosis was based on the severity of the screening result (Table 1). All tests results were tabulated locally and aggregate data were sent to the Ministry of Health.

**Table 1:** Classification of screening test results and recommendations made to individuals who participated in the National Campaign to Detect Diabetes Mellitus

		**Categories**^a^	**Recommendation**
Fasting capillary glucose^b ^(mmol/l)	< 5.6	Normal	Repeat test in 3 years
	5.6 to 6.0	High normal^c^	Schedule future appointment
	6.1 to 6.9	Borderline^c^	Schedule future appointment
	7.0 to 11.1	Altered	Order fasting serum glucose and recommend return medical appointment
	11.1 to 14.9	Diabetes likely	Order fasting serum glucose and schedule clinical appointment
	≥ 15.0	Diabetes very likely	Immediate consultation with physician
Non-fasting capillary glucose (mmol/l)	< 7.8	Normal	Repeat test in 3 years
	7.8 to 11.0	Borderline	Schedule future appointment
	11.1 to 14.9	Diabetes likely	Order fasting serum glucose and schedule clinical appointment
	≥ 15.0	Diabetes very likely	Immediate consultation with physician.

Brazil, 2001.

^a ^All screening results not classified as normal were considered positive.

^b ^Fasting was defined as absence of food ingestion 4 hours prior to capillary glucose test.

^c ^These subcategories were developed for the purpose of this study, having been treated as a single category (borderline) during the program implementation.

In all, 95.3% of the country's municipalities participated, with an estimated 31.1 million adults aged 40 or over being targeted for screening. In fact, a total of 22.1 million capillary glucose tests were performed.

To evaluate the initial impact of the screening program, a stratified sampling process was used to select first a random sample of 50 municipalities from all of Brazil's 5 regions, entrance probability being proportional to the number of screening tests reported, and second, in each municipality, one primary healthcare unit, again with probability proportional to the number of tests reported. For each of these units, individual screening forms obtained from the municipality were randomly sampled (random start with consecutive sampling thereafter) until an adequate number had been obtained, this goal initially set at 2,000 screenees and 200 positive screenees. In the event that a selected primary healthcare unit or municipality did not have 2,000 screenees or 200 positive screenees, additional forms from a contiguous primary healthcare unit or municipality (identified by an *a priori *scheme) were randomly selected in order to achieve the desired number.

A total of 126,376 individual forms from all screenees in the sampled primary healthcare units were thus selected. From this sample, 12,799 records were excluded because of missing data, illegibility, or improbable (< 2.2 and > 29.4 mmol/l) capillary glucose results. A final random sample of 1,996 records in each municipality was selected. In three municipalities, the number of records obtained approximated the original number desired (1,743; 1,833; and 1,895) to the point that additional forms from adjacent municipalities were not obtained. Two municipalities were excluded because more then 50% of the participants were recorded as having a positive screening test, which was considered more likely due to previous disposal of forms from negative individuals than to chance. From the remaining 95,291 records, 5,185 (5.4%, 95% CI 4.8 – 6.1%) indicating treatment for diabetes prior to screening were also excluded. Final analysis of data on screening forms thus considered 90,106 forms.

Home interviews were performed by trained professionals using a standardized and piloted questionnaire 15 to 19 months after screening. From the 200 positive screenees selected in each of the 48 municipalities, the first 100 were selected for interview. The remaining 100, in their original order of selection, served as a reserve in the event that those selected could not be located or refused interview.

In total, 7,183 individuals were actively procured, and 4,991 interviewed. Among the 2,192 participants not interviewed, 1,460 (67%) could not be located by the address provided during the campaign, 271 (12%) had moved, 202 (9%) were located but not found at home after three visits, 115 (5%) refused interview and 144 (7%) were not interviewed for miscellaneous reasons. As an additional 85 (1.7%) individuals had died by the time the interview, 4,906 positive screenees were actually interviewed.

Information obtained included socio-economic and lifestyle characteristics; underlying medical conditions; screening and confirmatory testing data and subsequent healthcare services received.

Data on diagnostic confirmation and subsequent health care from this sub-sample of positive screenees were extrapolated to estimate the number of cases of diabetes detected and the number of cases actually incorporated into the healthcare system overall as a result of the nationwide screening program. The number of individuals needed to screen to detect one case of diabetes and to incorporate one case into health care were also estimated.

Data was analysed using EpiInfo v.6.04d and SAS for Windows v.8 [19]. Confidence intervals for estimates derived from the sub-sample of 4,906 positive screenees interviewed in the follow-up study were estimated using STATA v.8 [20], with region being the stratification variable and municipal primary health care unit the cluster.

The Ministry of Health provided information on direct costs of the screening program at the national level, considering costs for mobilization and mass media advertising, purchase and distribution of screening test kits, training of healthcare workers, and overall screening program management.

The screening campaign was planned and conducted by personnel already working for the healthcare system. Considering opportunity costs associated with this campaign, labor costs were estimated considering the costs for individuals involved with planning and implementation of the campaign in the National, State, Municipal, and healthcare unit levels. Labor costs were estimated considering time allocation, professional category and respective salary ranges, and number of professionals involved in each of these levels. In the National level, 6 nurses and 4 medical doctors were working full time during 6 months (average monthly wage US$ 1,362 for nurse and US$ 1,915 for medical doctor) in planning and coordination of the screening campaign. In average 1 nurse was working full time during 2 months in all the 26 states and Federal District in planning and coordination in the State level (average monthly wage US$ 1,362). In all of the 5,301 municipalities participating in the screening campaign, 1 professional with a bachelor or higher degree was working during 2 months (average monthly wage US$ 1,064) in planning and coordination activities in the Municipal level. Time allocated by this professional to screening campaign activities was estimated as a function of the size of the municipality (full time in municipalities with population ≥ 500,000; half-time in municipalities ≥ 100,000 but <500,000; 25% in municipalities ≥ 50,000 but <100,000; and 10% in municipalities <100,000 population). One professional with a bachelor or higher degree was responsible for supervisory activities at the primary healthcare unit where capillary glucose testing was conducted. It was estimated that one such professional would have allocated in average 8 hours per week in each one of the 40,000 primary healthcare unit in the country during the campaign (average monthly wage US$ 1,064). In addition, community health agents were responsible for conducting the testing (average monthly wage US$ 269). It was estimated an average of 5 minutes per capillary glucose test performed to instruct campaign participants to answer the questionnaire and perform the testing, totaling 110,349,525 minutes or 1,839,159 hours of work of these professionals in the local level (22,069,905 tests performed * 5 minutes).

As confirmatory diagnosis consumes additional resources, these costs were estimated for individuals who tested positive during the screening Campaign and reported having returned for diagnostic confirmation (37.1%). These costs included the costs of a confirmatory fasting plasma glucose test and two additional physician visits for each individual. For confirmation through the national healthcare system, reimbursement values of US$ 0.79 for the glucose test and US$ 1.09 for each physician visit were used to estimate these costs [21]. Confirmatory diagnosis through the private sector was estimated considering reference reimbursement values from the Brazilian Medical Association [22], these being US$ 1.49 for a fasting plasma glucose test and US$ 5.32 for each physician visit.

Professionals who interviewed and screened participants provide regular services at the primary healthcare units and are paid at the municipal level. A questionnaire was sent to a convenience sample of 14 municipalities selected for their known strong engagement in the screening program in order to evaluate the amount of costs incurred at the local level during the nationwide screening program. Direct municipal costs such as additional costs with media, logistics and social mobilization, were estimated as an additional proportion of total national costs.

This analysis considers the public healthcare system perspective since the costs of the nationwide screening program are paid by this system. Time horizon considered was 1 year and no costs or benefits were discounted.

All costs are presented in US Dollars, considering the exchange rate of the Brazilian Real (R$) to the US Dollars (US$) in December 2001 (1 US$ = 2.35 R$). To allow for international comparison, results are also presented in international dollars (Int$) considering the purchasing power parity exchange rates in 2001 (1 Int$ = R$ 0.59), as recommended by the WHO [23]. For sensitivity analyses, based on results of the convenience sample, we estimated that additional local costs during the nationwide screening program could have ranged from 10–25% of the total costs dispensed by the national level.

A decision analytic model was used to estimate cost per new diabetes case diagnosed as a result of the nationwide screening program. Screening campaign was compared to no screening. Costs per case were obtained dividing total costs by the estimated number of cases diagnosed and by the estimated number of cases incorporated into health care.

We estimated the impact of variation in characteristics of the screening program on the estimated cost per diabetes case diagnosed through one-way sensitivity analysis. Characteristics included proportion of individuals in a fasting state when screened, proportion of individuals with known diabetes among those screened, proportion of positive screenees returning for confirmatory diagnosis, and proportion of confirmatory testing done at public vs. private providers. Additional local costs and a range of varying labor costs during screening was also included in sensitivity analysis. Base case scenario estimates and ranges for each parameter included in sensitivity analysis are presented in Table 2.

**Table 2:** Parameters considered in base case analysis and range for selected parameter estimates included in sensitivity analysis

**Parameters**	**Base Case Estimate**	**Range**
Proportion of population ≥ 40 years who participated in the screening program	73%	-
Percentage of tests in screenees who reported to be under treatment for diabetes prior to screening	5.4%	4.8 – 6.1^a^
Percentage of subjects in fasting state when screened^b^	46.7%	30 – 50%^c^
Percentage of positive screening tests^d^	16.4%	-
Percentage of positive screenees who reported having diabetes diagnosis prior to the screening program^e^	16%	14 – 18.1%^a^
Percentage of positive screenees who returned for confirmatory testing	37.1%	0 – 100%^c^
Percentage of positive screenees who were diagnosed as having diabetes mellitus	10.1%	-
Percentage of positive screenees diagnosed with diabetes and incorporated into the healthcare system	9.4%	-
Additional local costs	0%	10 – 25%^f^
Estimated labor costs	US$ 5.99 million	US$ 4.65 – 8.98 million
Percentage of positive screenees who returned for confirmatory testing in the public system (as opposed to the private health sector)	100%	75 – 25%^c^

National Campaign to Detect Diabetes Mellitus. Brazil, 2001.

^a ^Range corresponds to the 95% confidence interval of each estimate from the probabilistic sample.

^b ^Fasting was defined as absence of food ingestion 4 hours prior to capillary glucose test.

^c ^Range corresponds to an arbitrary estimate of the authors.

^d ^Fasting glucose ≥ 5.6 mmol/l or a casual glucose ≥ 7.8 mmol/l.

^e ^These subjects knew their previous diabetes diagnosis; however, they had not provided that information when asked about it during screening program.

^f ^Range expressed as an additional percentage of national costs.

This study was carried out in accordance with the Declaration of Helsinki as revised in 2000 [24]. The project was approved by the Ethics Committee of the Universidade Federal do Rio Grande do Sul. Written informed consent was obtained prior to interview.

### Ethical Approval

The study was approved by the ethics committee of the Federal University of Rio Grande do Sul, Brazil.

## Results

Evaluation of the 90,106 screening forms revealed 16.4% of screening exams to be positive.

Of the sample of 4,906 positive screenees interviewed, 56.9% were women, 47.3% were aged 60 and older, and 81.2% had less than a complete primary school education. Although interviews were performed only for individuals not reporting treatment for diabetes at screening, surprisingly, 786 (16%, 95% CI 14 – 18.1%) of those interviewed reported having known diabetes prior to screening. Additionally, 394 individuals (8%, 95% CI 5.8 – 10.2%) reported not remembering having participated in the screening program at interview, approximately 1 1/2 years afterwards. Of the remaining 3,726 individuals, 1,821 (48.9%, 95% CI 45.1 – 52.8%) informed that they had returned for confirmatory diagnostic testing. This proportion increased notably with increasing capillary glucose value at screening (See Additional file 1).

The most frequent test used for diagnostic confirmation was fasting venous glucose (1,399, 76.8%), followed by fasting capillary glucose (310, 17%). Of these tests, 1,282 (70.4%) were publicly financed, 275 (15.1%) being covered by medical insurance and 239 (13.1%) paid for out-of-pocket. The average time elapsed from screening to confirmatory testing was 3 months. Medical consultation for diagnostic confirmation followed testing in 371 (79%) of these cases; the time elapsed from confirmatory testing to consultation was less than 8 days for half of the patients, and ≤ 20 days for 75%. Of the medical visits, 265 (71.3%) were to public providers; with 212 (57.5%) being at a primary healthcare unit.

The proportion of screen positives confirming a diagnosis of diabetes varied according to screening result, from 1.6% (95% CI 0.9 – 2.8%) for those with a screening fasting glucose of 5.6–6.1 mmol/l to 59.7% (95% CI 52.7 – 66.4%) for those with a screening glucose ≥ 15.0 mmol/l) (See Additional file 1). In the sample analysed, 28 individuals had diabetes diagnosis established at screening, and 469 at return visit by confirmatory test. Thus, the total number of newly diagnosed diabetes cases in the sample was 497, of whom 458 (92.2%) reported to be receiving medical treatment at a healthcare service when interviewed. Of those diagnosed, 210 (42.3%) were aged 60 years and older, and 403 (81.1%) had less than a complete primary education. Self-reported data on weight and height indicated overweight (BMI ≥ 25 kg/m^2^) in 294 (59.2%) and low-weight (BMI <18.5 kg/m^2^) in 113 (22.7%) of these newly diagnosed diabetes cases. In addition, 41 (8.8%) of the individuals who underwent confirmatory testing reported a result characterized by the physician as not diabetes but "sugar intolerance" (thus likely being diagnosed with impaired glucose tolerance or impaired fasting glucose).

Among the 786 individuals who participated in the screening program but at interview reported diabetes diagnosis prior to screening, 355 (45.2%) were not receiving medical care for diabetes prior to screening. Of these, 93 (26.2%) reported diabetes diagnosis confirmation after screening and 88 (24.8%) reported to be receiving medical care for diabetes at interview.

**Figure 1:**
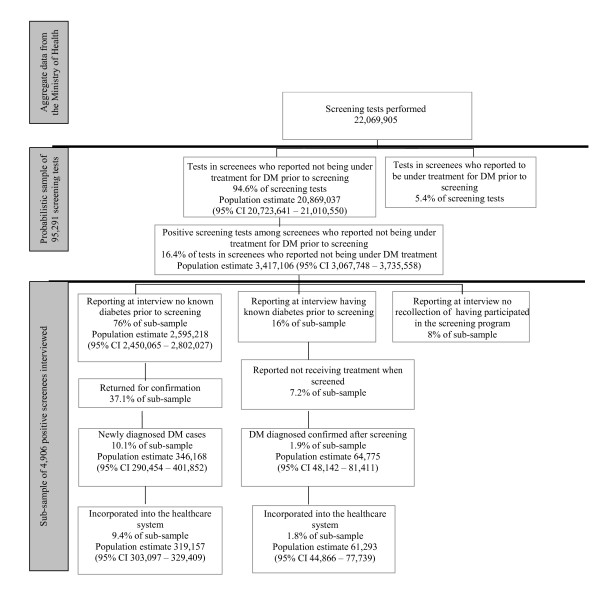
Population estimates for the initial impact of the National Campaign to Detect Diabetes Mellitus. Brazil, 2001.

Figure 1 outlines our estimate that 346,168 (95% CI 290,454 – 401,852) new diabetes cases were diagnosed, of which 319,157 (95% CI 303,097 – 329,409) were incorporated into healthcare. Thus, the number of screening tests needed to detect one new case of diabetes was 64 (95% CI 55 – 76). As an additional 61,293 (95% CI 44,866 – 77,739) individuals with previous diabetes diagnosis but not receiving medical assistance prior to screening also reported linking to medical care, a total of 380,450 (95% CI 322,917 – 437,731) cases of diabetes were incorporated into the healthcare system as a result of the screening program. Given this additional incorporation of prevalent but untreated cases, the number of tests needed to incorporate one new case into the health system was 58 (95% CI 50 – 68).

Total direct program costs dispensed at the national level for screening were US$ 22,423,155 (Int$ 89,312,567). Purchase of diagnostic material, including reagent strips and glycometers, was the largest cost item in the nationwide screening program – US$ 13.19 million. Costs of reagents and glycometers were US$0.42 per target individual to be screened ($13.19 million/31.1 million population). Costs of personnel involved in planning and implementing screening campaign were estimated at US$ 5,988,783 (Int$ 23,853,627).

Total mobilization costs were US$ 3.11 million. Mobilization costs considered were: elaboration of a media plan; media time in radio, television, newspaper and billboard announcements used to publicize screening days; and printed material used during the Campaign (posters, booklets, forms etc). Total training and program management costs were US$ 136,499. Training on the use of capillary glycometers was provided by the manufacturer as part of the purchase contract.

Confirmatory diagnostic costs consumed an additional US$ 3.75 million, as an estimated 1.26 million individuals returned for diagnostic confirmation at an estimated cost of US$ 2.97 per person. Total cost for the screening (US$ 22.4 million) and diagnostic confirmation was thus US$ 26.19 million.

Assuming base case scenario (Table 2), the cost per new diabetes case diagnosed was US$ 76 (Int$ 301). The cost per diabetes case incorporated into healthcare, considering previously untreated individuals as well as newly diagnosed cases, was US$ 69 (Int$ 274).

In sensitivity analysis, varying the proportion of individuals with prior diabetes diagnosis participating in the screening program did not impact significantly the estimated number of new diabetes cases diagnosed. However, varying the proportion of those fasting when screened did, as the fasting cut point chosen (5.6 mmol/l), though lacking in specificity, was more sensitive than the non-fasting one (7.8 mmol/l); 32.7% of those fasting tested positive, as opposed to only 8.9% of those having eaten in the past 4 hours. If 50% of participants had been fasting instead of 31%, using these cut points, the number needed to screen to detect 1 case would have decreased from 64 to 54.

Additionally, if a higher percentage of positive screenees had returned for confirmation, higher screening effectiveness would have been achieved, reaching 36 diabetes cases diagnosed per 1,000 screened (if 100% had returned). In this case, cost per diabetes case diagnosed would decrease to US$ 53 (Int$ 212).

When considering additional local level costs, cost per case detected increased proportionally, reaching US$ 80 (if 10%) and US$ 88 (if 25%). In the base case scenario, we assumed that all positive screenees who returned for confirmatory testing did so at the public health sector. If 25% or 75% of positive screenees had confirmed diagnosis with private providers, the cost per case detected would increase 11% (US$ 84) and 33% (US$ 101), respectively.

Likewise, when labor cost estimates were considered as 50% higher than base case (reaching US$ 8.98 million), cost per case detected increased to US$ 84. Considering that it is unlikely that a professional with a bachelor or higher degree would have been available for supervisory activities in all 40,000 primary healthcare units in the country, we estimated labor costs assuming such professionals were available in only 10,000 primary healthcare units in the country. In such scenario estimated personnel costs would be US$ 4.65 million and cost per case detected increased to US$ 72.

## Discussion

### Principal findings

This massive population screening program conducted via public healthcare clinics of Brazil, a large middle income country, identified 3.4 million individuals as having positive screening tests for diabetes. After diagnostic confirmation, nearly 350,000 new cases of diabetes were detected (one per 64 screened). Although the primary objective of a screening program is to detect undiagnosed cases of diabetes, some known cases of diabetes not previously receiving treatment were also incorporated through the Campaign. Thus, a slightly larger number of cases (one per 58 screened) were incorporated into the health system. The cost per new case of diabetes diagnosed was US$ 76.

### Strengths of the study

Several features of the diabetes screening program contributed to its success in terms of participation and yield, and deserve comment. First, the screening was an integral part of a major reorganization plan focusing on primary healthcare services in which early detection of diabetes was not an isolated objective. Since training primary care providers was one of the plan's main activities, those who conducted the screening were generally motivated. The provision of diagnostic follow-up and care generally took place at the site of screening.

Second, as diabetes awareness was an important aspect of the plan, mass communication strategies were successful in achieving a high participation rate [17].

Third, the Brazilian primary care network, like many in low and middle income countries, has accumulated years of experience in vaccination campaigns occurring in a concentrated format over a short period of time. In this setting, a "campaign approach" for diabetes detection was seen by many as a natural extension of these preventive activities.

Finally, positive screenees received a scaled recommendation to seek diagnostic confirmation, priority being given to those with greater hyperglycemia. This avoided patient overload during the initial months of the Campaign and ensured that diagnosis and treatment would be provided to those with greater probability of having diabetes. This approach did, however, contribute to the low rate of confirmatory testing and thus a relatively low proportion of confirmed diabetes among screen positives (See Additional file 1), especially among those in the lower range of hyperglycemia.

Considering the need to improve quality of care for individuals with diabetes in Latin America [25], a significant contribution of this program was its ability to use screening as a cornerstone of a larger plan aimed at reorganizing the care of diabetes. The benefit of this approach is illustrated by the additional incorporation of approximately 60,000 cases of diabetes previously aware of their disease yet apparently outside the reach of the health sector.

Although overall nationwide screening costs were significant, the cost per new case of diabetes diagnosed was lower than that observed in community screening strategies reported in the literature, described as ranging from US$ 100 to US$ 741 [26-30]. It should be noted, however, that cross-national comparisons of program costs are difficult to interpret as these depend heavily on labor costs, resource utilization, and other costs which are country specific. Relative to developed countries reported high labor cost, lower labor costs is likely to have influenced the screening campaign total costs and cost per diagnosed case in Brazil.

### Implications

Screening for diabetes remains a controversial issue. Early detection and treatment of diabetes logically allows for early implementation of interventions proven to reduce morbidity and mortality associated with diabetes and its complications. Yet, universal screening for diabetes is not recommended, mainly due to the lack of convincing evidence that the benefits of early detection and treatment of undiagnosed diabetes [11,31] justify the additional costs incurred. Most guidelines recommend selective opportunistic screening [13,32] of patients seeking healthcare for other reasons.

Undeniable gains in the organization of primary care resulted from the inclusion of screening in the Brazilian National Plan for the Reorganization of Diabetes Mellitus and Hypertension Care. In so doing, millions were mobilized across the country, thus demonstrably placing diabetes on the agenda of primary care professionals. The intangible nature of these gains and their associated costs makes it difficult to contextualize the initial benefits and costs of this unique effort with those of other reported population-based screening efforts.

Several countries employ periodic public "campaigns" to optimize prevention of infectious diseases through vaccination. To date, similar public campaigns for chronic disease prevention, such as screening for diabetes, have not been conducted and evaluated. This campaign approach can, at relatively low cost, mobilize the population and health system, suggesting its potential use in diabetes prevention in selected settings.

One possibility, in this regard, would be to join diabetes screening, or other chronic diseases prevention initiatives with influenza vaccination campaigns.

### Study limitations

Several limitations should be noted. The quality and legibility of information recorded on screening forms filled out by busy practitioners during the Campaign and the exclusion of 2 of the 50 municipalities due to incomplete records led to considerable missing information. The difficulty of locating addresses and frequent change of address in less privileged settings in Brazil compounded this problem. How this might have biased our findings is not clear. If less privileged members of the community were in fact more likely to be so excluded, our study may underestimate true yield and thus overestimate true cost per case, as these individuals would have been more likely to have unknown diabetes, given inadequate pre-Campaign access to health care. Additionally, information regarding confirmatory testing and follow-up care were based on self-reports given by screening participants during interviews conducted one year later, rather than on actual confirmatory testing, leading to inaccuracies. Finally, as an oral glucose tolerance test was rarely used for the diagnosis of diabetes in the clinical setting in Brazil in 2001, many cases of diabetes by isolated 2 h hyperglycemia were missed by the Campaign.

## Conclusion

This nationwide population-based screening program, conducted through primary care services, demonstrates the feasibility, within the context of an organized national healthcare system of a middle income country, of conducting screening campaigns for chronic disease. Although overall costs were significant, cost per new case diagnosed was comparable or lower than those previously reported. However, cost-effectiveness analysis and considerations of the strategic value of a campaign approach are necessary before recommending this form of screening for other settings or for repeated use in Brazil.

## Competing interests

The authors declare that they have no competing interests.

## Authors' contributions

Cristiana M. Toscano, Sotero S. Mengue, Luciana B. Nucci, Carisi Anne Polanczyk, Bruce B. Duncan and Maria Ines Schmidt participated in the design of the evaluation research project, data collection, statistical analysis and manuscript drafting. Claudio D. Fonseca and Adriana Costa e Forti participated in the overall implementation of the Brazilian National Screening Campaign to Detect Diabetes Mellitus, as well as in the supervision and design of its evaluation. They reviewed results and the manuscript.

## Pre-publication history

The pre-publication history for this paper can be accessed here:



## Supplementary Material

Additional file 1 **Main initial impact of the screening program, as estimated from the follow-up study, sub-sample of the National Campaign to Detect Diabetes Mellitus. Brazil, 2001.** The data provided represent the impact results of the screening program, as estimated from the follow-up study, sub-sample of the National Campaign to Detect Diabetes Mellitus. Brazil, 2001. Click here for file
