# Supplementary material for: Initial impact and cost of a nationwide population screening campaign for diabetes in Brazil: A follow up study
Source: BMC Health Serv Res. 2008 Sep 22;8:189. doi: 10.1186/1472-6963-8-189 (PMC2562380; doi:10.1186/1472-6963-8-189)
Supplement: Additional file 1 — Main initial impact of the screening program, as estimated from the follow-up study, sub-sample of the National Campaign to Detect Diabetes Mellitus. Brazil, 2001. The data provided represent the impact results of the screening program, as estimated from the follow-up study, sub-sample of the National Campaign to Detect Diabetes Mellitus. Brazil, 2001. [file 1472-6963-8-189-S1.doc]

Main initial impact of the screening program, as estimated from the follow-up study, sub-sample of the National Campaign to Detect Diabetes Mellitus. Brazil, 2001.

| Screening Results | Positive screenees | | Confirmatory tests done | | Confirmed diabetes cases | | Cases of diabetes incorporated into healthcare | |
| --- | --- | --- | --- | --- | --- | --- | --- | --- |
|  | N | % | N | % ^a^ (95% CI ) | N | %^a^ (95% CI ) | N | % ^b^ (95% CI ) |
| High normal | 739 | 19.8 | 214 | 29.0 (24.2 – 34.4) | 12 | 1.6 (0.9 – 2.8) | 9 | 75.0 (46.1 – 91.3) |
| Borderline | 1974 | 53.0 | 957 | 48.5 (44.6 – 52.7) | 125 | 6.3 (5.1 – 7.9) | 116 | 92.8 (84.6 – 96.8) |
| Altered | 417 | 11.2 | 219 | 52.5 (48 – 57.2) | 75 | 18.0 (14.2 – 22.5) | 68 | 90.7 (81.2 – 95.6) |
| Diabetes likely | 303 | 8.1 | 201 | 66.3 (60.4 – 71.8) | 110 | 36.3 (30.6 – 42.4) | 101 | 91.8 (82.6 – 96.4) |
| Diabetes very likely | 293 | 7.9 | 230 | 78.5 (72.9 – 83.2) | 175 | 59.7 (52.7 – 66.4) | 164 | 93.7 (88 – 96.8) |
| Total | 3726 | 100 | 1821 | 48.9 (45.1 – 52.8) | 497 | 13.3 (11.2 – 15.5) | 458 | 92.2 (87.6 – 95.1) |

95% CI = 95% confidence interval

^a^ Percent of positive screenees in the given screening result category.

^b^ Percent of confirmed diabetes cases in the given screening result category
